# Polyisoprenylated cysteinyl amide inhibitors disrupt actin cytoskeleton organization, induce cell rounding and block migration of non-small cell lung cancer

**DOI:** 10.18632/oncotarget.15956

**Published:** 2017-03-07

**Authors:** Elizabeth Ntantie, Jerrine Fletcher, Felix Amissah, Olufisayo O Salako, Augustine T Nkembo, Rosemary A Poku, Francis O Ikpatt, Nazarius S Lamango

**Affiliations:** ^1^ College of Pharmacy and Pharmaceutical Sciences, Florida A&M University, Tallahassee, Florida 32307, USA; ^2^ University of Miami Miller School of Medicine, Miami, Florida 33136, USA

**Keywords:** RhoA, cell invasion, filopodia, lamellipodia, PCAIs

## Abstract

The malignant potential of Non-Small Cell Lung Cancer (NSCLC) is dependent on cellular processes that promote metastasis. F-actin organization is central to cell migration, invasion, adhesion and angiogenesis, processes involved in metastasis. F-actin remodeling is enhanced by the overexpression and/or hyper-activation of some members of the Rho family of small GTPases. Therefore, agents that mitigate hyperactive Rho proteins may be relevant for controlling metastasis. We previously reported the role of polyisoprenylated cysteinyl amide inhibitors (PCAIs) as potential inhibitors of cancers with hyperactive small GTPases. In this report, we investigate the potential role of PCAIs against NSCLC cells and show that as low as 0.5 μM PCAIs significantly inhibit 2D and 3D NCI-H1299 cell migration by 48% and 45%, respectively. PCAIs at 1 μM inhibited 2D and 3D NCI-H1299 cell invasion through Matrigel by 50% and 85%, respectively. Additionally, exposure to 5 μM of the PCAIs for 24 h caused at least a 66% drop in the levels of Rac1, Cdc42, and RhoA and a 38% drop in F-actin intensity at the cell membrane. This drop in F-actin was accompanied by a 73% reduction in the number of filopodia per cell. Interestingly, the polyisoprenyl group of the PCAIs is essential for these effects, as NSL-100, a non-farnesylated analog, does not elicit similar effects on F-actin assembly and organization. Our findings indicate that PCAIs disrupt F-actin assembly and organization to suppress cell motility and invasion. The PCAIs may be an effective therapy option for NSCLC metastasis and invasion control.

## INTRODUCTION

Lung Cancer remains the leading cause of cancer-related deaths in both men and women in the United States [1]. More people die of lung cancer each year than colon, breast and prostate cancers combined [1]. About 85% of all lung cancer cases are of the non-small cell lung cancer (NSCLC) histological type [2]. NSCLC accounts for the majority of lung cancer deaths because of its metastatic potential [1]. Metastatic NSCLC has a poor prognosis, with a 5-year survival rate of 4% [1].

The Rho family of small GTPases have been implicated in the development and progression of lung cancer [3]. The Rho proteins are ubiquitously expressed in most cell and tissue types. These proteins are intricately involved in the organization and assembly of the F-actin cytoskeleton and thus regulate cellular processes of adhesion, migration and invasion [4]. These cellular processes are commonly dysregulated during cancer development and progression to drive tumor metastasis and invasion.

Members of the Rho family of proteins that have been implicated in NSCLC development and progression include RhoA [5], RhoB [6, 7], RhoC [8, 9], Cdc42 [10] and Rac1 [11, 12]. The Rho proteins are rarely mutated in cancers, but rather their overexpression and/or hyper activation is responsible for promoting the malignant phenotype in most cancers. Overexpression of RhoA, RhoC and Cdc42 proteins has been reported to promote the aggressive phenotype of NSCLC [5, 8, 9]. Overexpression of Rac1 [11, 12] or in some instances, alternative splicing of Rac1 to generate oncogenic Rac1b [13] has been reported to enhance NSCLC malignancy. On the contrary, RhoB has been reported to suppress the aggressive phenotype of NSCLC [6, 7, 14] and its downregulation was shown to correlate with lung tumor progression [6].

The Rho proteins are key regulators of actin dynamics and thus control F-actin assembly and organization [4, 15, 16]. The organization of F-actin is characterized by the formation of dynamic structures such as stress fibers, filopodia and lamellipodia [4, 16]. Stress fibers are important and complex bundles of F-actin fibers that extend throughout a whole cell and connect the cell to the extracellular matrix through focal adhesions points [16, 17]. Filopodia are protruding extensions from cells formed from the assembly of 15-20 actin filaments into bundles [18]. These structures are important for probing the environment and for propelling a cell during cell migration [19]. Lamellipodia are flat extensions that are generated at the leading edges of migrating cells. These structures are thought to result from the formation of branched and compact bundles of F-actin [19].

The formation of stress fibers, filopodia and lamellipodia are distinct events triggered by the activation of different members of the Rho family of small GTPases. The activation of the Rho small GTPase, for example RhoA, is characterized by the formation of stress fibers. The activation of Cdc42 is accompanied by the formation of filopodia, whereas the activation of Rac1 is characterized by the formation of lamellipodia or membrane ruffles [4, 16]. However, crosstalk between members of the Rho Family of GTPases has been reported to result in the concerted formation of lamellipodia, filopodia and/or stress fibers on the same cell [4, 16]. It is therefore not surprising that NSCLC exhibit increased expression or activation of the Rho small GTPases to augment F-actin remodeling, and drive the migratory potential of metastatic NSCLC [3]. Of concern are the limited therapeutic options for patients with metastatic NSCLC that justifies the need for novel and innovative therapies.

We previously reported the synthesis of polyisoprenylated cysteinyl amide inhibitors (PCAIs) for testing as potential anti-cancer agents [20]. PCAIs were designed to target polyisoprenylated methylated protein methyl esterase (PMPMEase), an enzyme which is overexpressed and hyperactive in lung cancer cells and tissues [20, 21]. We demonstrated that PCAIs are poor inhibitors of PMPMEase but potent inhibitors of cell viability, cell proliferation, and apoptosis in pancreatic cancer cells [20]. The effects of PCAIs on Rho protein function, F-actin assembly and organization, cell migration and invasion have not yet been demonstrated in NSCLC. In this study, we investigate the role of PCAIs on Rho localization, lamellipodia and filopodia formation, F-actin assembly, cell morphology on NSCLC migration and invasion. Our results demonstrate that micro-molar concentrations of PCAIs diminish Rho protein levels and an F-actin-binding protein from the plasma membrane in a time-dependent manner; disrupt filopodia and lamellipodia formation in a time-dependent manner; disrupt F-actin assembly, promote cell rounding, and inhibit 2D and 3D NSCLC migration and invasion. These results strongly point to a role of PCAIs in disrupting the functions of RhoA and F-actin in cell motility and invasion. PCAIs thus have the potential to be developed as anti-metastatic agents for the treatment of NSCLC.

## RESULTS

### The PCAIs, NSL-BA-040, suppresses NSCLC cell monolayer and spheroid migration

**Figure 1:**
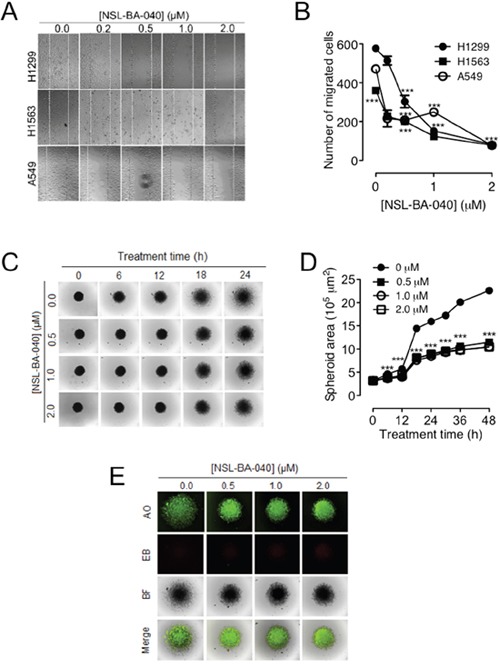
NSL-BA-040 suppresses lung cancer cell migration in 2D and 3D cultures **(A)** Confluent monolayers of lung cancer H1299, H1563, and A549 cells each separated by a “wound” generated using cell culture inserts (ibidi) were treated with the indicated concentrations of NSL-BA-040 and closure of the wounds was monitored and images captured using a Nikon *Ti* Eclipse microscope at 4X magnification. **(B)** The numbers of cells that migrated into the wounds were counted. The results are the means of at least three independent experiments. **(C)** H1299 cells were seeded into 96-well Nunclon Sphera plates at a density of 1.0 × 10^4^ cells/well to form spheroids. After 48 h, the compact spheroids were transferred onto 8-well chamber glass bottom plates coated with gelatin and incubated for an additional 24 h to allow cells to attach to gelatin. This was followed by treatment with NSL-BA-040 and the migration of cells from the spheroid body was monitored every 6 h for 48 h. **(D)** Time-dependent effect of NSL-BA-040 on spheroid area. **(E)** The observed effect of NSL-BA-040 on spheroid area is not due to apoptosis as shown by AO/EB staining. The green fluorescence of AO indicates that the cells are viable and the lack of EB staining indicates that the concentrations of NSL-BA-040 (0 -2.0 μM) used are not cytotoxic under the study conditions. Statistical significance (***p < 0.001) was determined using 1-way ANOVA with post hoc Dunnett's tests.

Previously, we reported that micro-molar concentrations of NSL-BA-040 inhibited migration of pancreatic cancer cells in wound healing assays [22]. We next tried to see if NSL-BA-040 would have a similar effect on the migration of NSCLC cells. To characterize the effect of NSL-BA-040 on NSCLC cell migration, we examined the ability of NSCLC cells to migrate from a confluent monolayer into an area devoid of cells over a 24 -36 h period. Treatment with NSL-BA-040 (0.2 – 2.0 μM) resulted in cells repopulating the cell-free area at a slower rate compared to control cells (Figure 1A). The rate of migration was dependent on the concentration of NSL-BA-040 and decreased significantly with increase in its concentration. For all cell lines tested, we observed a significant decrease in migration with as little as 0.5 μM NSL-BA-040. At this concentration, the migration of H1299 was suppressed by 48%, H1563 by 44% and A549 by 56% (Figure 1B). Higher concentrations further reduced NSCLC cell migration by 74% in H1299, 65% in H1563 and 50% in A549 cells with 1 μM treatment, and 87% in H1299 cells, 78% in H1563 and 83% in A549 cells with 2 μM treatment of NSL-BA-040 (Figure 1B). Overall, the migration of H1299 cells was the most inhibited of the NSCLC cell lines tested.

Although monolayer cell migration is commonly used for migration studies, one of its pitfalls is that tumor do not grow as monolayers but rather in 3D [23, 24]. To simulate a more physiological model of migration, we examined the ability of H1299 cells to migrate on gelatin from an H1299 spheroid body (Figure 1C). We generated 2-day old H1299 spheroids of average diameter 640 ± 30 μm, placed these on gelatin-coated plates, and monitored the migration of cells from the spheroid using live cell imaging microscopy. As expected, exposure to NSL-BA-040 slowed the migration of cells on gelatin as compared to the controls (Figure 1C). Exposure to 0.5 μM NSL-BA-040 resulted in a 45% and 54% reduction in migration after 24 and 48 h of treatment, respectively. To determine if the concentrations of NSL-BA-040 used were cytotoxic to spheroids, we stained spheroids with an AO/EB (5 μg/mL) solution (Figure 1E). Spheroids took up AO but not EB indicating that the cells were viable at all concentrations of NSL-BA-040 used. These results demonstrate that the observed effects are due to inhibition of cell migration rather than cytotoxicity.

### PCAIs suppress 2D and 3D NSCLC cell invasion

**Figure 2:**
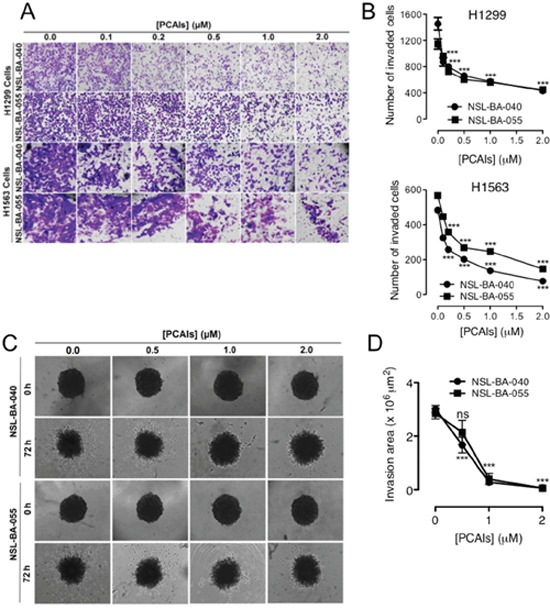
PCAIs suppress 2D and 3D cell invasion **(A)** NSCLC cells (H1299 and H1563) were plated onto the inserts of 24-well Matrigel invasion chambers and incubated for 24 h as indicated in the methods. Cells that invaded from the top chamber of inserts through Matrigel were trapped on a membrane on the lower chamber of the inserts. These invading cells were fixed with a 7:1 mixture of methanol to acetic acid and then stained with 1% crystal violet. Bright field images were obtained using an Olympus IX70 Microscope. **(B)** Each treatment condition performed in triplicates was quantified by counting the number of NSCLC cells that invaded using the NIS-Element software. **(C)** Preformed H1299 spheroids were embedded into 5 mg/mL Matrigel containing acetone only or PCAIs in 8-replicates and incubated (5% CO_2_; 37°C) for 1 h. After 1 h, the Matrigel had solidified and treatment media (RPMI + 5% FBS) containing equivalent concentrations of PCAIs were then added on top of the Matrigel. Bright field (BF) images of spheroids were immediately acquired (0 h) using a Nikon *Ti* microscope at 4X magnification. Images at 4X magnification were subsequently acquired at 72 h. **(D)** The effect of the PCAIs on invasion by the H1299 cells was determined by measuring the area invaded by cells from the spheroid body using the NIS-Element software. Statistical significance (***p < 0.001) was determined using 1-way ANOVA with post hoc Dunnett's tests.

For cells to metastasize, in addition to their migration from a primary tumor, they need to invade through the extracellular matrix to proximal and distal tissues. To better understand the potential ability of the PCAIs to inhibit NSCLC cell metastasis, trans-well invasion assays were used. We observed a concentration-dependent decrease in the number of cells that invaded through Matrigel following exposure to PCAIs (NSL-BA-040, NSL-BA-055) (Figure 2A). Exposure to 1 μM of PCAIs diminished invasion of H1299 cells by 50% compared to control (Figure 2B) while 2 μM of NSL-BA-040 and NSL-BA-055, further decreased invasion of H1299 to 70% and 61%, respectively (Figure 2B). A similar decrease in invasion was observed with the H1563 cell line, where 1 μM of the PCAIs diminished cell invasion by 56% and 72% with exposure to NSL-BA-040 and NSL-BA-055 respectively (Figure 2B). NSL-BA-040 and NSL-BA-055 inhibited 74% and 84% of H1563 cell invasion at 2 μM, respectively (Figure 2B). Although 2D cell invasion assays are very informative, again tumors grow in 3D thus the need to recapitulate an *in vitro* tumor model of invasion [24]. For these assays, premade H1299 spheroids of average diameter 1070 ± 30 μm (Supplementary Figure 1) were embedded into Matrigel supplemented with the PCAIs. The ability of cells to break free from a spheroid and invade their surrounding was monitored by time-lapse microscopy (Figure 2C). In these assays, we observed a dramatic reduction in the area of invasion when the PCAIs were added to Matrigel. Matrigel-containing 1 μM of either NSL-BA-040 or NSL-BA-055 resulted in 91% and 86% reduction in invasion area, respectively (Figure 2D). Furthermore, Matrigel-containing 2 μM of PCAIs obliterated the ability of the H1299 cells in spheroids to invade Matrigel (Figure 2D). Together, our results demonstrate that the PCAIs effectively block invasion of NSCLC through Matrigel.

### PCAIs inhibit cell survival, colony formation and formation of viable spheroids

**Figure 3:**
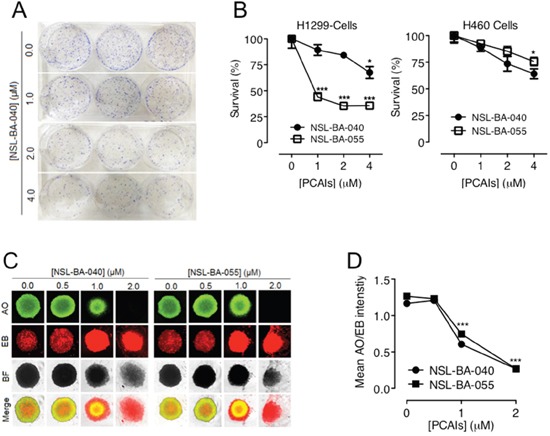
PCAIs inhibit NSCLC cell survival, colony and spheroid formation **(A)** NSCLC cells plated at a density of 1 × 10^3^ cells/well in 6-well plates were treated in triplicates with the indicated concentrations of the PCAIs. Identical amounts of PCAIs were used to supplement the samples after 24 h for a 48 h exposure. After 48 h incubation, the cells were harvested, washed twice with PBS and re-plated at 0.5 x10^3^/well and 1×10^3^/well in complete media (RPMI + 10% FBS) without PCAIs and incubated (5% CO_2_, 37°C) for an additional 12 - 14 days. The resulting colonies were fixed with a 7:1 mixture of methanol to acetic acid and stained with 1% crystal violet. Digital images of stained colonies were captured as shown. **(B)** The number of colonies were manually counted and plotted as shown. Significance (*p< 0.05; ***p < 0.001) was determined by 1-Way ANOVA followed by Dunnett's post-hoc test. **(C)** H1299 cell suspensions (5.0 × 10^4^ cells/mL) in treatment media (RPMI + 5% FBS) containing the indicated concentrations of PCAIs were seeded into 96-well Nunclon Sphera plates and incubated for 24 h. Identical amounts of PCAIs were used to supplement the samples after 24 h for the 48 h treatment. After 48 h incubation, spheroids were stained with AO/EO (5 μg/mL). Fluorescent and BF images were then captured at 4X magnification using Nikon *Ti* Eclipse microscope. **(D)** Concentration-dependent decreases in mean intensity ratios of AO/EB were determined for each treatment concentration performed in 8 replicates. The mean intensity ratios AO/EB denote the ratio of viable to non-viable cells in the spheroids. Statistical significance (***p < 0.001) was established using 1-way ANOVA followed by post hoc Dunnett's tests.

To begin to understand the application of PCAIs to prevent tumor relapse, we investigated the ability of NSCLC cells to survive, grow and form colonies or spheroids after exposure to PCAIs. We used H1299 cells in these assays since, as shown in Figures 1 and 2, the PCAIs elicit robust effects on migration and invasion in this cell line. Additionally, we used the H460 cell line that tends to form good colonies. In these assays, using H1299 cells, we observed that NSL-BA-040 had a modest effect on cell survival and colony formation. Prior exposure of H1299 cells to 4 μM NSL-BA-040 for 48 h, followed by re-plating of the cells in the absence of NSL-BA-040, staining, and counting of colonies after 12-14 days revealed a decrease in survival to 68% (Figure 3A, 3B). Exposure to lower concentrations (1 μM and 2 μM) of NSL-BA-040 did not significantly inhibit colony formation. In contrast, NSL-BA-055 markedly reduced colony formation in H1299 cells at all concentrations tested (Figure 3B). Exposure to 1 μM NSL-BA-055 resulted in 44% colony survival, whereas 2 or 4 μM of NSL-BA-055 diminished colony survival down to 35% (Figure 3B). The H460 cell line demonstrated a more modest response to NSL-BA-055 when compared to the H1299 cell line. There was no significant reduction in colony survival for H460 cells that were pre-exposed to lower concentrations of both PCAIs (1 μM, 2 μM), whereas pre-exposure to 4 μM significantly decreased the survival of H460 colonies to 65% (Figure 3B). These results indicate that the PCAIs had a concentration-dependent effect on colony formation, with NSL-BA-055 being more potent than NSL-BA-040. Additionally, H1299 cells were more susceptible to the PCAIs in these assays than the H460 cells.

We next wondered if pretreatment of the H1299 cells with the PCAIs would attenuate their ability to form viable spheroids. To test this possibility, we designed spheroid formation assays where H1299 cells were pre-treated with varying concentrations of PCAIs (0.0-2.0 μM) and then allowed to form spheroids in the presence of the PCAIs. While the H1299 cells formed spheroids under these conditions, however, the viability of the spheroids significantly decreased with increasing concentrations of PCAIs (Figure 3C). The PCAIs were comparably potent in these assays in rendering H1299 cells susceptible to apoptosis. Exposure to 1 μM and 2 μM of NSL-BA-040 led to the formation of spheroids that were 52% and 23% viable, respectively, while exposure to 1 μM and 2 μM of NSL-BA-055 generated spheroids that were 59% and 21% viable, respectively (Figure 3D). These results demonstrate that pre-treatment of NSCLC cells with PCAIs renders cells susceptible to cell death by apoptosis, thereby diminishing colony formation and spheroid viability.

### PCAIs induce polyisoprenylation-dependent changes in the morphology of H1299 cells

**Figure 4:**
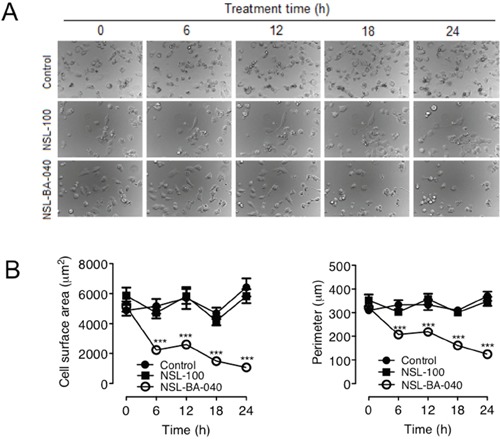
NSL-BA-040-induced changes in H1299 cell morphology are dependent on the farnesyl moiety **(A)** H1299 cells (1.0 × 10^4^ cells/well) were plated onto the 15-μ-slide 4-well chamber glass bottom plates (*ibidi*) and incubated overnight. The following day, the cells in one well were treated with acetone (1 μl), whereas the cells in the other wells were treated with either 5 μM of NSL-BA-040 or NSL-BA-100 each contained in 1 μl of acetone. Immediately following treatment, the cells were placed in a microscope stage incubator (37 °C/ 5% CO_2_) and DIC images were captured at 6 h intervals for 24 h using a Nikon *Ti* Eclipse microscope at 30X magnification. Representative DIC Images are shown. **(B)** The effects of the compounds on cell morphology were determined by measuring the surface areas and perimeters of cells in the captured images using the NIS-Element software (N = 100). The control group was determined to be statistically significant from NSL-BA-040 treatment group by 1-way ANOVA, with post hoc Dunnett's test. Statistical significance at the different time points was then determined by Student's t-test (***p < 0.001).

Migratory cells are morphologically dynamic cells that undergo time-dependent changes in cell shape and size as they attach and detach to their substratum [25]. In contrast, less migratory or non-migratory cells are usually rounded-up [25]. In our quest to better understand the role of PCAIs in cell migration, we examined the time-dependent changes in size of H1299 cells. In addition to using the PCAIs in these assays, we synthesized an analog of the PCAIs, NSL-100 in which the farnesyl moiety is replaced with an ethyl group [26]. Using live cell imaging microscopy, we captured DIC images of cells at different time points after exposure to 5 μM NSL-BA-040 or 5 μM of NSL-100 (Figure 4A). As expected, we observed that treatment with NSL-BA-040 induced rounding of cells and markedly reduced cell surface areas and/or cell perimeters. Treatment with NSL-BA-040 resulted in a 57% decrease in cell surface area at 6 h; 71% decrease at 12 h and 79% decrease at 24 h (Figure 4B). Interestingly, NSL-100 did not induce cell rounding and did not significantly alter the cell surface areas and perimeters of the H1299 cells (Figure 4A, 4B). Put together, our results indicate that the NSL-BA-040 induces cell rounding and decreases in cell surface areas and importantly these effects are dependent on the farnesyl moiety of the PCAIs.

### PCAIs decouple RhoA/F-actin intensity profiles, induce vesicle formation, loss of F-actin and disruption of filopodia

**Figure 5:**
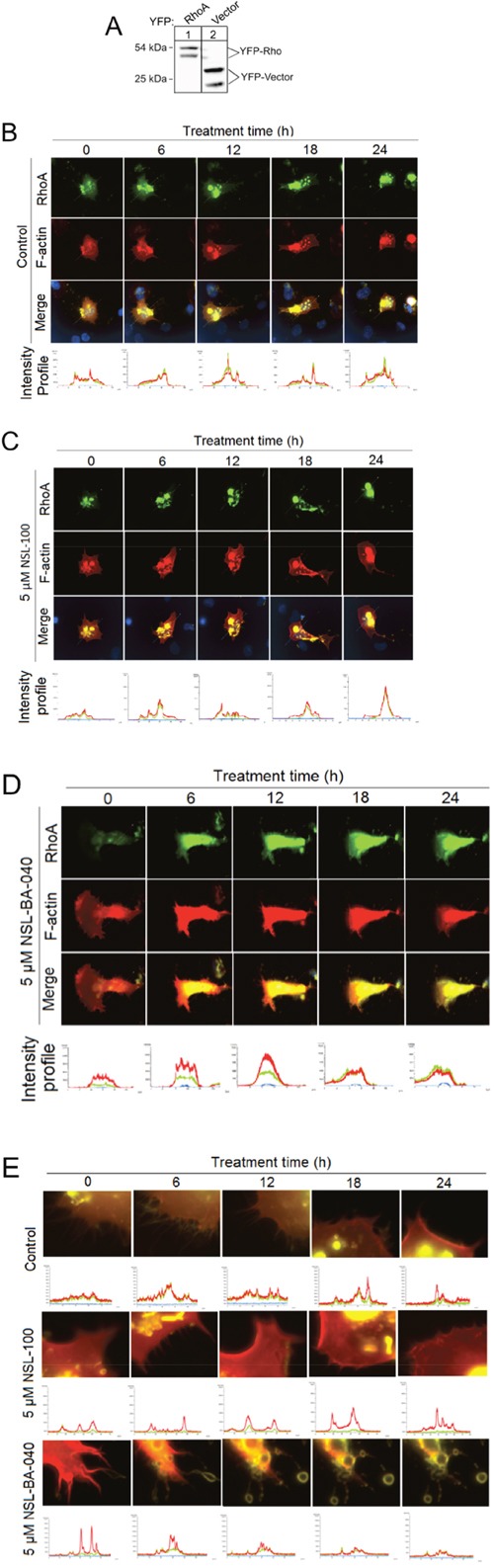
PCAIs decouple RhoA/F-actin intensity profiles, induce exocytosis and loss of F-actin and disrupt Filopodia in H1299 cells **(A)** Lysates generated from H1299 cells overexpressing YFP-RhoA or YFP-vector were analyzed by Western Blotting using Living Colors RCFP antibody (Clontech) and HRP-conjugated rabbit IgG secondary antibody to visualize the expression of YFP-Rho proteins and YFP-vector as shown. **(B)** H1299 cells (1.0 × 10^4^ cells/mL) transiently co-expressing YFP-Rho A and RF-actin-BP were plated onto 15-μ-slide 4-well chamber glass bottom plates and incubated overnight. The next day, cells were placed into a microscope-stage incubator (37°C/5% CO_2_). Cells were treated either with 1 μl of acetone and fluorescent images captured every 6 h for a 24 h period using the Nikon *Ti* Eclipse microscope at 60X magnification. Fluorescence intensity profiles were generated using the NIS-Element software. **(C)** Similar to “B” except that cells were treated with 5 μM NSL-100 dissolved in 1 μl of acetone. **(D)** Similar to “B” except that the cells were treated with or 5 μM NSL-BA-040 dissolved in 1 μl of acetone. Note loss of F-actin and decoupling of intensity profiles at 18 and 24 h. **(E)** Images “B –D” but with focus at the cell membrane. Notice NSL-BA-040 induces a time-dependent formation of vesicles, loss of F-actin intensity at the membrane, and disruption of filopodia.

Since the Rho family of GTPases are intricately involved in cell migration, invasion and spreading, we next examined the effect of NSL-BA-040 on RhoA and F-actin in H1299 cells co-expressing YFP-RhoA and RF-actin-BP proteins. First, we confirmed the expression of YFP-RhoA in these cells by western blotting of whole cell lysates using a polyclonal antibody RCFP antibody (Clontech) directed against YFP. In western blots, we observed two immuno-reactive proteins in lysates expressing YFP-RhoA. These immuno-reactive proteins consisted of a slower migrating protein at 54 kDa and a faster migrating protein at 40 kDa on SDS PAGE (Figure 5A, lane 1). Similarly, in control lysates expressing the YFP-vector only, we observed a slower migrating immuno-reactive protein at 32 kDa and a faster migrating immuno-reactive protein at 24 kDa (Figure 5A, lane 2). Because the RCFP-antibody (Clontech) is a polyclonal antibody, it is very likely that the bands at 40 kDa and 24 kDa (lanes 1 and 2, respectively) are non-specific bands, as YFP-RhoA is expected to run at 54 kDa and YFP-vector at 32 kDa on SDS-PAGE. At this juncture, it is important to state that the antibody product sheet from Clontech similarly shows two immuno-reactive proteins in cell lysates expressing the YFP-Vector and probed with this antibody.

After confirming the expression of YFP-RhoA in the H1299 cells, we used live image microscopy to study the time-dependent changes in RhoA and F-actin. YFP-RhoA was localized to the cell membrane and to endo-membranes of the cell (Figure 5B). RF-actin-BP, which binds specifically to F-actin, was observed at the plasma membrane and cytoplasm. While there was no significant observable temporal change in RhoA and F-actin intensity profiles in both the control and NSL-100-treated cells (Figure 5B, 5C), exposure to NSL-BA-040 (5 μM) induced a time-dependent loss in cytoplasmic material and greatly compromised the integrity of the cell (Figure 5D). These findings suggest that NSL-BA-040 compromises the cell membrane thereby inducing loss of either RhoA and/or F-actin.

To better understand the effects of NSL-BA-040 on the RhoA and F-actin cytoskeleton, we re-examined the images of H1299 cells co-expressing YFP-RhoA and RF-actin-BP, this time focusing our attention on the cell membrane. Interestingly, we observed a time-dependent loss in F-actin intensity but not YFP-RhoA intensity at the membrane (Figure 5E) after exposure to NSL-BA-040. The loss of F-actin intensity was revealed by examining the temporal fluorescence intensity of RFP (red)/YFP (green) ratios. Whereas the temporal fluorescent intensity ratio (TRITC/YFP) remains unchanged in control and NSL-100 treatment groups, it drops by 53% at 18 h and 24 h with exposure to NSL-BA-040. This drop in F-actin fluorescent intensity is accompanied by the disruption of filopodia (Figure 5E) and pinching off of F-actin-containing vesicles (Figure 5E). These data indicate that exposure to NSL-BA-040 induces loss of F-actin but not RhoA from these H1299 cells and that the loss of F-actin occurs concomitantly with disruption of filopodia and release of F-actin-containing vesicles from cell membranes.

### PCAIs disrupt F-actin organization to inhibit filopodia and lamellipodia formation

**Figure 6:**
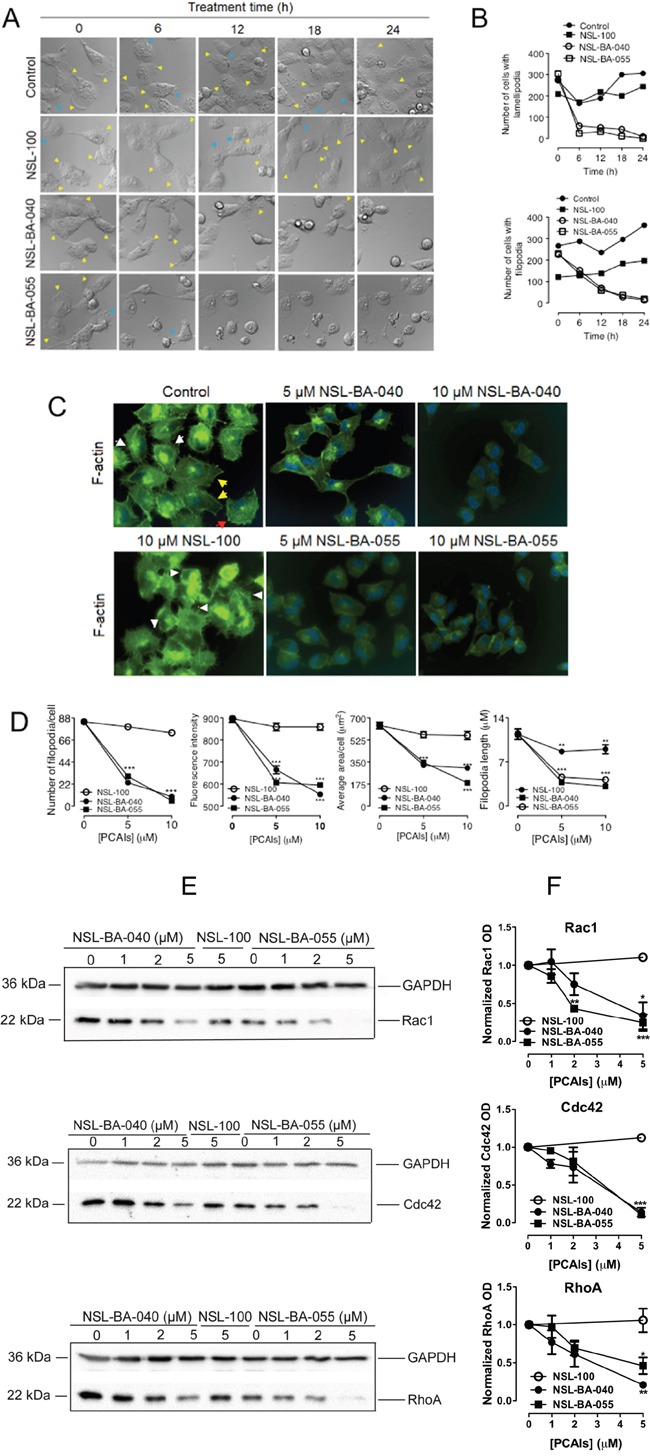
PCAIs diminish levels of Rho GTPases to disrupt filopodia and lamellipodia **(A)** H1299 cells (1.0 × 10^4^ cells/well) transiently co-expressing YFP-RhoA and a RF-actin-BP were seeded into 15-μ-slide 4-well chamber plates (ibidi) and incubated (5% CO_2_; 37°C) for 24 h. Cell media were replaced with treatment media (5% FBS) containing either 0.1% acetone (vehicle), PCAIs (5 μM NSL-BA-040 and 5 μM NSL-BA-055) or 5 μM NSL-100. DIC images were immediately obtained (0 h) and then subsequently acquired every 6 h for a 24 h using the Nikon Ti Eclipse microscope at 60X magnification; lamellipodia and filopodia are indicated by the yellow and blue arrowheads, respectively. **(B)** The number of cells with lamellipodia and/or filopodia were counted and graphed (N = 400 cells). **(C)** H1299 cells (1.0 × 10^4^ cells/well) were plated onto sterile glass coverslips that were placed into the wells of a 24-well plate. The cells were incubated overnight to allow them to attach onto the coverslips before they were treated with acetone (control), PCAIs (5 μM, 10 μM), or NSL-100 (5 μM, 10 μM) for 24 h. After 24 h treatment, cells were washed with PBS and fixed onto the coverslips using 3.7% formaldehyde solution, permeabilized with 0.1% Triton X-100, and stained with Phalloidin-iFluor 488 (Abcam) to detect for F-actin fibers. Coverslips were then mounted onto microscope slides with mounting media containing DAPI. Cells were visualized at 40X using a Nikon *Ti* Eclipse microscope; filopodia are indicated by the white arrowheads, lamellipodia by the red arrowheads and stress fibers by the yellow arrowheads. **(D)** The concentration-dependent change in the number of filopodia per cell, filopodia length, the F-actin fluorescent intensity (FITC), and cell area of phalloidin-iFluor-488 stained cells that were pretreated with either PCAIs (5 μM, 10 μM) or NSL-100 (5 μM, 10 μM) are as shown in the graphs (N = 50 cells).**(E)** Lysates generated from H1299 cells exposed to PCAIs (0 -5 μM), or 5 μM NSL-100 for 24 h were analyzed by Western Blotting using antibodies against Rac1, Cdc42, and RhoA (Cell Signaling Technology, Danvers, MA) and HRP-conjugated rabbit IgG secondary antibody to visualize the levels of the Rho proteins. **(F)** Quantification of Rho protein levels in E. Data are representative of three independent experiments. Statistical significance (**p < 0.01, ***p< 0.001) was determined by 1-way ANOVA with post hoc Dunnett's test.

The observation that NSL-BA-040 induces loss of F-actin, accompanied by the disruption of filopodia in H1299 cells, as shown in Figure 5 suggests that the PCAIs attenuate filopodia and lamellipodia formation. Filopodia are formed by cells to probe the environment before lamellipodia are formed for directional motility [19]. To confirm the role of the PCAIs in F-actin organization, we captured DIC time-lapse images of H1299 cells exposed to 5 μM of the PCAIs, NSL-100 or 0.1 % acetone (Figure 6A). We then quantified the number of cells exhibiting either filopodia or lamellipodia as an indirect measure of the extent of F-actin organization. As expected, we observed a dynamic change in the number of cells exhibiting lamellipodia and/or filopodia in control and NSL-100 treated cells over a 24 hour time period. The overall time-dependent change in the number of cells with lamellipodia and/or filopodia followed a similar pattern in the control and NSL-100 groups (Figure 6B). In marked contrast, exposure to PCAIs led to significant reduction in the number of cells with lamellipodia and/or filopodia. We observed a 79% and 92% drop in the number of cells with lamellipodia, 6 h after exposure to NSL-BA-040 and NSL-BA-055, respectively (Figure 6B). At 24 h post-treatment with the PCAIs, there were no cells with lamellipodia. Additionally, we observed a drop in the number of cells with filopodia following exposure to PCAIs, albeit at a much slower rate. The number of cells exhibiting filopodia, reduced by 33% and 42%, 6 h after treatment with NSL-BA-040 and NSL-BA-055, respectively. At 12 h post-treatment, we observed a 74% and 88% drop in the number of cells with filopodia following NSL-BA-040 and NSL-BA-055 treatment, respectively. At 24 h post treatment, NSL-BA-040 induced a 94% loss in the number of cells with filopodia, whereas NSL-BA-055 induced a 92% loss. These data are in agreement with previous results (Figure 5) demonstrating a time-dependent loss of F-actin at the cell membrane.

### PCAIs disrupt F-actin organization and assembly

To further characterize the roles of PCAIs on the F-actin cytoskeleton organization, we treated H1299 cells with 5 μM and 10 μM PCAIs for 24 h, fixed the cells and then stained them with Phalloidin-iFluor-488. Phalloidin-iFluor-488, which specifically binds to F-actin, allowed for the fluorescent visualization of F-actin organization in these cells (Figure 6C). Several features depicting F-actin organization in the cells included filopodia (white arrowheads), lamellipodia (red arrowheads) and stress fibers (yellow arrowheads) as shown in Figure 6C. Interestingly, the H1299 cells had more filopodia than any of the other two elements. To determine the extent by which the PCAIs affected F-actin organization, we quantified the number of filopodia/cell, the fluorescent intensity, the average cell surface areas, and the average filopodia lengths. As shown in Figure 6D, the PCAIs dramatically diminished the number of filopodia/cell from 84 in control cells to 23 and 30 in cells treated with 5 μM NSL-BA-040 and NSL-BA-055, respectively. Treatment with 10 μM PCAIs further decreased the number of filopodia/cell to 9 and 5 with NSL-BA-040 and NSL-BA-055 treatments, respectively (Figure 6D). PCAIs also impeded the elongation of filopodia as the average length of filopodia decreased by at least 60% and 65% with exposure to 5 μM and 10 μM of PCAIs, respectively (Figure 6D). Similarly, we observed a concentration-dependent decrease in the fluorescent intensities and cell surface areas following exposure to the PCAIs. The PCAIs caused up to 38% and 71% drop in fluorescence intensity and cell surface areas, respectively (Figure 6D). In contrast, NSL-100 did not significantly alter the number of filopodia/cell, the fluorescent intensity and cell surface area but moderately attenuated the length of filopodia by 24% and 21% at 5 μM and 10 μM exposures, respectively (Figure 6D). These results provide strong evidence that the PCAIs disrupt F-actin organization and assembly thereby inducing cell rounding. While the farnesyl moiety is required for disrupting F-actin organization, assembly and cell rounding, its presence worsens filopodia length elongation.

### PCAIs diminish Rac1, Cdc42, and RhoA protein levels to disrupt F-actin organization

The Rho GTPases are key regulators of F-actin organization and remodeling [4, 15, 16]. Our findings that the PCAIs disrupt the organization of F-actin led us to predict that the PCAIs may be interfering with the levels and/or activation of Rac1, Cdc42 and RhoA proteins. We investigated the first possibility by analyzing lysates generated from H1299 cells following 24 h treatment with the PCAIs, or NSL-100. In these assays, we observed a significant drop in the levels of all Rho GTPases examined after 24 h exposure to 5 μM PCAIs (Figure 6E, 6F). The protein levels of Rac1 dropped by 66% and 75%; Cdc42 levels dropped by 86% and 90%, while RhoA levels dropped by 80% and 55% when the cells were exposed to 5 μM NSL-BA-040 and NSL-BA-055, respectively. As expected, 5 μM of the non-farnesylated NSL-100 did not alter the levels of Rac1, Cdc42 and RhoA.

Put together, we provide compelling evidence that the PCAIs diminish Rac1, Cdc42 and RhoA protein levels to block the organization, remodeling and assembly of the F-actin cytoskeleton into filopodia and lamellipodia structures, thereby abrogating cell migration and invasion. Importantly, the farnesyl moiety is required for the effects of the PCAIs in disrupting F-actin cytoskeleton.

## DISCUSION

The actin and microtubule cytoskeleton play an important role in tumor invasion and metastasis as they are involved in cellular processes such as cell adhesion, cell cycle progression, angiogenesis, cell morphology and cell motility [27]. Cancer cells augment the remodeling of their cytoskeletons to promote their proliferative, invasive and migratory phenotypes. Taxanes, which disrupt microtubule dynamics, have been widely used in the clinic as chemotherapeutic agents in the treatment of cancers [28]. Several attempts to similarly disrupt the actin cytoskeleton have not been successful. Cytochalasin D and jasplakinolide that bind directly to actin show high toxicity in preclinical testing as potential anti-cancer agents [29–31]. A logical explanation for this is that these agents bind directly to actin filaments thereby poisoning cells. Developing agents that modulate the F-actin cytoskeleton, by disrupting the functions of their upstream and/or downstream effectors, should be beneficial in our quest for effective and targeted cancer therapies. In this study we describe for the first time, the role of PCAIs, a novel class of compounds that were synthesized to mimic the posttranslational modifications in polyisoprenylated small monomeric G-proteins, in modulating the F-actin cytoskeleton to suppress lung cancer cell migration and invasion. In a recent report [26], we demonstrated that the PCAIs possess anti-angiogenic effects. We report that the PCAIs (1) suppress 2D and 3D NSCLC migration and invasion; (2) induce morphological changes promoting cell rounding; (3) disrupt F-actin organization and diminish filopodia density and (4) diminish Rac1, Cdc42 and RhoA protein levels but do not alter RhoA localization to compromise their functions in cytoskeletal organization. These findings suggest that the PCAIs possess the potential to be developed as effective therapeutic agents that modulate the actin cytoskeleton.

Cell migration and invasion are cellular processes that drive tumor metastasis. Consequently, effective treatments against tumor metastasis would require agents that suppress cell migration and invasion. Although 2D models of migration and invasion are widely used and informative, these models fall short in recapitulating what happens *in vivo* as tumor cells do not grow in monolayers. Signaling between cells in 2D is expected to be different from those in 3D cultures. We observed that the PCAIs significantly suppress the migration of NSCLC cells from spheroids on gelatin, the substance of the ECM. Additionally, invasion of NSCLC cells from spheroids through Matrigel was abrogated upon exposure to the PCAIs. Importantly, the effects of the PCAIs on migration and invasion were observed at concentrations that are non-cytotoxic. These results point to the potential of the PCAIs to be effective therapies in suppressing metastasis at concentrations that are not cytotoxic thereby eliminating the potential toxic side effects. The PCAIs-induced effects against cell migration and invasion, coupled with the aforementioned potent effects against angiogenesis [26] imply that metastasis is likely to be severely limited in the presence of the PCAIs.

Prompted by the finding that the PCAIs suppress NSCLC cell migration and invasion, we wondered if the PCAIs might alter the morphology of NSCLC cells given that the cytoskeletal structures for cell motility are also involved in the maintenance of cell shape. Cells respond to changes in their environment by changing their shapes and sizes. Migratory cells tend to spread out as they form new attachments to the ECM whereas non-migratory cells become more rounded as they retract their membrane protrusions and cell margins [32–34]. Analysis of cell spreading or rounding is typically performed by the quantification of cell surface area and/or perimeter [35]. As expected, the PCAIs induced temporal rounding of NSCLC cells by diminishing their surface areas and perimeters. These observations were the same in assays utilizing both live and fixed NSCLC cells after exposure to the PCAIs. These findings support the role of the PCAIs in inducing morphological changes that attenuate cell motility.

Changes in cell morphology are usually preceded by changes in the F-actin cytoskeleton. The F-actin cytoskeleton imparts mechanical and structural characteristics to cells. F-actin organization during cell motility typically involves the formation of filopodia and then lamellipodia at the leading edges of cells [4, 19, 27]. Filopodia are initially formed to “survey” the environment before lamellipodia are formed for directional movement [36]. We hypothesized that the PCAIs disrupt F-actin organization that then lead to changes in cell motility and morphology. The observed time-dependent suppression of filopodia and lamellipodia formation and the decrease in the number of filopodia/cell and filopodia length has profound clinical implications since filopodia density has been reported to correlate with tumor prognosis [36]. High numbers of filopodia are linked to invasive carcinomas [36]. A decrease in the number of filopodia/cell suggests disruption in filopodia initiation, whereas a decrease in the length of filopodia suggests disruption in its elongation. It is possible that the PCAIs disrupt filopodia-related proteins such as fascin and formins since fascin and formins regulate F-actin nucleation and elongation, and are strongly implicated in cancer metastasis and invasion [36–39]. Interestingly, NSL-100 moderately attenuated filopodia length albeit to a lesser extent than the PCAIs. These findings indicate that the farnesyl moiety is required and essential for the PCAIs to disrupt F-actin organization and impede filopodia elongation.

Filopodia and lamellipodia formation are tightly regulated processes controlled by several polyisoprenylated proteins including the Rho family of small monomeric proteins such as Rac1, RhoA and Cdc42 [40]. Our findings that the PCAIs disrupt F-actin organization suggest that the PCAIs may attenuate the levels and/or activity of these RhoGTPases. We examined this possibility and observed that the PCAIs did indeed significantly diminish the levels of Rac1, Cdc42 and RhoA proteins. The expression and stability of the Rho GTPases has been reported to be regulated by post-translational modifications, GDP/GTP exchange and formation of protein complexes through their interactions with regulators such Guanine nucleotide Exchange Factors (GEFs) [41]. The PCAIs may thus alter one or several of the above mentioned processes in order to diminish the levels of Rac1, Cdc42, and RhoA. During filopodia and lamellipodia formation, the leading edge of a migrating cell is enriched with phosphatidylinositol (PI) lipids; PI(3,4,5)-triphosphate, PI(3,4)-bisphosphate, and PI(4,5)-bisphosphate [36]. These membrane-anchored lipids contain a pleckstrin homology (PH) domain that provides docking sites for several PH-domain proteins. A plethora of proteins are then recruited to the cell membrane by PI lipids. These include the WASP family of proteins which are important for lamellipodia formation, Myosin-X which is important for the filopodia formation [36, 42], and Rho GEFs [43, 44]). These PH-containing proteins interact with the PI lipids and the polyisoprenylated Rho GTPases to accomplish their functions in F-actin cytoskeleton organization and thus the formation of filopodia or lamellipodia. It is thus not surprising that elevated levels of PI lipids have been reported to promote metastasis in several cancer types [45]. Since the PCAIs mimic the polyisoprenylation modifications of GTPases, they may either insert themselves into the plasma membrane to disrupt the accumulation of the PI lipids at the membrane or disrupt Rho GTPase interactions with their binding partners. The latter is most likely the case as most of the proteins recruited by the PI lipids to the leading edge interact with polyisoprenylated Cdc42 or Rac1 to stimulate filopodia or lamellipodia formation. For instance, the PCAIs could bind onto the Rho GEF, Vav that contains a PH-domain. Such binding will impede the formation of a signalosome and thus activation of several signaling molecules such as Myosin-X and WASP thereby retarding the formation of filopodia and lamellipodia. This line of reasoning is consistent with Miao's (et al.) report [46] indicating that small molecules disrupt PI (3,4,5)-triphosphate binding to the PH-domain of several proteins.

When we co-expressed YFP-RhoA and RF-actin-BP in NSCLC cells, we noticed that while exposure to the PCAIs did not alter the localization of the YFP-RhoA protein, they decoupled the apparent co-localization of both proteins as depicted by changes in the relative intensities of the fluorescence profiles of YFP-RhoA and RFP-F-actin, with that of RFP-F-actin significantly decreasing at the cell membrane. This loss in F-actin is concomitant with the formation of F-actin-containing vesicles that could be observed pinching off the cells upon treatment. The role of F-actin remodeling during exocytosis is well documented [47–50] and our observations are consistent with reports that F-actin is lost at the plasma membrane during exocytosis in secretory cells [51, 52]. It appears that loss of F-actin that is signified by the decrease in RFP-F-actin intensity may explain for the decoupling of RhoA and F-actin intensity profiles. Our observation that the PCAIs do not alter the localization of YFP-RhoA suggests that the PCAIs do not block protein polyisoprenylation, or dislodge the Rho GTPases from the plasma membrane since RhoA localization is dependent on its polyisoprenylation [53, 54]. The PCAIs-induced loss of filopodia and lamellipodia most probably results from the decrease in Rho GTPase protein levels, loss of F-actin at the membrane, and decoupling of YFP-Rho and RFP-F-actin. The inability of NSL-100 to elicit similar effects on cell morphology, Rho GTPase protein levels, RhoA/F-actin decoupling, filopodia and lamellipodia disruption validates the importance and requirement of the farnesyl moiety of PCAIs in attenuating F-actin cytoskeleton remodeling.

The success of any anticancer agent in eradicating metastasis will be short-lived unless it can prevent tumor reoccurrence. Tumor relapse is thus a major concern in the development of anticancer agents. Our results indicate that the PCAIs diminish anchorage-dependent formation and survival of colonies. Consistent with these results, we additionally observed that pre-exposure to PCAIs renders NSCLC cells susceptible to apoptosis even at low concentrations that did not otherwise induce apoptosis in these cells. Taken together, these findings suggest that the PCAIs may have prophylactic effects at low concentrations. This is important as some lung cancers that go into remission with the current treatment options, reoccur. These cancers are usually more aggressive resulting in poorer prognosis for the affected patients.

In conclusion, our findings uncover a new and innovative approach to treating metastasis by modulating the F-actin cytoskeleton. The benefits of pharmacologically controlling the F-actin cytoskeleton in the treatment of tumor metastasis are significant as cytoskeletal remodeling is involved in multiple cellular processes and events such as in EMT that drive the transformed phenotype. Although more work is needed to fully decipher the precise mechanism by which the PCAIs elicit their effects, nonetheless our previous findings that the PCAIs attenuate angiogenesis [26] and current observations on cell migration and invasion make them a desirable candidate to be developed for the targeted treatment of lung cancers with hyperactive signaling pathways involving polyisoprenylated proteins. We are currently conducting structural and biochemical assays to identify the molecular target(s) to which the PCAIs bind to in order to elicit their F-actin modulating effects. It is important to mention that the present findings are not only important for potential lung cancer therapy but also for other cancers that exhibit aberrantly high activities of Rho proteins. These include breast, pancreatic, prostate and colon cancers where F-actin remodeling is a common phenomenon that promotes tumor invasion and metastasis.

## MATERIALS AND METHODS

### Cell culture

All NSCLC cell lines; NCI-H1299, NCI-H1563, NCI-H460 and A549 were purchased from ATCC (Manassas, VA). The cell lines H1299, H1563 and H460 were maintained in RPMI-1640 medium with 10% heat-inactivated fetal bovine serum (FBS) and antibiotics; the A549 cell line was maintained in F-12K medium with 10% heat-inactivated fetal bovine serum and antibiotics. All cell lines were treated in base media (RPMI-1640 or F-12K) containing 5% FBS which is referred to here as treatment or experimental media. The PCAIs, NSL-BA-040, NSL-BA055, and their non-farnesylated analog NSL-100 were used for treatment. The chemical structures are as previously described [26].

### cDNA constructs

A cDNA encoding for the human RhoA protein was synthesized, cloned into the pZsYellow1-C1 vector (Clontech, Mountain View, CA), and sequenced for verification at GENEWIZ (South Plainfield, NJ). cDNA encoding for RFP-aRFctin-BP (pCMV-LifeAct-TagRFP) was purchased from ibidi (Madison, WI).

### Transient transfections

NSCLC cells were plated at 60% confluency and transiently transfected with plasmids using Lipofectamine 3000 reagent per manufacturer's instructions.

### Enhanced chemiluminescence (ECL)-western blotting

H1299 cells that were transfected with YFP-RhoA or the YFP-vector (pZsYellow1-C1) were lysed in RIPA buffer, boiled in Laemmli sample buffer and subjected to SDS-PAGE. The proteins were transferred onto polyvinylidene difluoride (PVDF) membrane and immunoblotted using an antibody against pZsYellow (Living Colors Anti-RCFP polyclonal pan antibody) from Clontech (Mountain View, CA). Bound antibodies were visualized using horseradish peroxidase-linked anti-rabbit IgG (Santa-Cruz Biotechnology, sc-2004) and ECL reagents (Bio-Rad) per manufacturer's recommendation.

### PCAIs diminish levels of Rac1, Cdc42 and RhoA in ECL-western blotting

H1299 cells were plated in RPMI media containing 10% FBS at a density of 5.0 × 10^4^/mL into each 100 × 20 mm tissue culture dish. The cells were incubated (37°C/5% CO_2_) overnight to allow them to adhere to the plates. The next day, the media were removed and replaced with experimental media containing PCAIs (0 – 5 μM) or 5 μM NSL-100 and then incubated for 24 h. The cells were washed with PBS, lysed with RIPA buffer and the amount of protein in lysates was determined using Pierce BCA Protein Assay kit (Thermo Scientific, Rockford, IL). Lysate volumes containing 50 μg of protein were boiled in Laemmli sample buffer and subjected to SDS-PAGE. Proteins were then transferred onto PVDF membrane and immunoblotted using antibodies against Rac1, Cdc42 and RhoA (67B9) that were purchased from Cell Signaling Technology (Danvers, MA). Bound antibodies were visualized using horseradish peroxidase-linked anti-rabbit IgG (Santa-Cruz Biotechnology, sc-2004) and ECL reagents (Bio-Rad) according to the manufacturer's recommendation.

### Live cell imaging

H1299 cells transiently co-expressing YFP-Rho and RFP-actin-BP proteins were plated into 15-μ-slide 4-well chamber plates at a cell density of 1.0 × 10^4^ cells/mL. After 24 h, the adhered cells were then treated with either 5 μM of NSL-BA-040, 5 μM NSL-BA-055, 5 μM NSL-100 or 0.1% acetone (vehicle) before fluorescent or DIC images were captured using a Nikon *Ti* Eclipse microscope at either 30X or 60X magnifications. To minimize bleed-through from the yellow and red fluorophores, the emission wavelength for detecting YFP was set at 520 nm and that of RFP at 650 nm.

### Wound healing assays

Cell culture inserts purchased from ibidi were used to generate two confluent monolayers of cells separated by a “wound” for these assays. This was achieved by placing NSCLC cells (2 - 4 × 10^5^ cells/mL) into each side of the ibidi-cell culture inserts attached onto the wells of a 24-well plate. The plate was then incubated (37°C/5% CO_2_) overnight to allow the cells to attach onto the plate and form two adherent confluent monolayers of cells on either side of the tissue culture insert. The next day, the cells were serum-starved by replacing complete media with base media (RPMI 1640 or F-12K) and then further incubated for 24 hours. After serum starving, the insert was gently removed to generate a gap or “wound’ between the two confluent layers of cells. The monolayers were washed with experimental media once and then fresh experimental media containing varying concentrations of PCAIs (0 -2 μM) were added. Bright-field microscope images around the “wound” were captured every 6 h until control wounds had closed. The number of cells that migrated into the “wounds” were counted for control and treated cells. Data were analyzed using GraphPad Prism 5.0.

### Spheroid migration on gelatin

Suspensions of H1299 lung cancer cells (2.5 x10^4^ cells/mL) were seeded into 96U Nunclon Sphera plates (Thermo Scientific, Waltham, MA) and incubated (37°C/5% CO_2_) for 48 h to form compact spheroids. A protocol described by Vinci et al. [55] was then adapted. Briefly, 8-well chamber cover glass plates (Lab-Tek, Rochester, NY) were incubated with a sterile 0.1% gelatin solution overnight at 37°C/5% CO_2_. The next day, gelatin solution was removed and the plates were left to dry under sterile conditions. After the 8-well chamber plates had dried out, 900 μL of treatment media containing either 1 μL acetone, 0.5 μM, 1.0 μM or 2.0 μM NSL-BA-040, each in 1 μL acetone, were added to different wells of the plate in quadruplicates. One spheroid in 100 μL was then transferred into each well of the 8-well chamber plates. At this time, the plates were incubated (37°C/5% CO_2_) for 24 h to allow the spheroids to attach to gelatin. After the spheroids had attached to gelatin, the migration of H1299 cells from the spheroid body was captured every 6 h for a total time period of 24 h. To ascertain the viability of the cells, spheroids were stained with 5 μg/mL of Acridine Orange/Ethidium Bromide (AO/EB) solution, which stains the nuclei of live and death cells green and red, respectively. Time-dependent changes in spheroid areas were measured for each concentration of NSL-BA-040 (0.0 - 2.0 μM) using the NIS-Element software. Data was analyzed in GraphPad Prism 5.0.

### Monolayer cell invasion assay

NSCLC cells suspensions (2.0 × 10^5^ cells / mL) in RPMI medium containing 0.1% FBS and varying concentrations of PCAIs (0 -2 μM) were placed into the upper chambers of 24-well plate Matrigel invasion inserts (Corning, Bedford, MA) that had been previously rehydrated with DMEM medium for 1 h at 37°C/5%CO_2_. To the wells of the 24-well plates was added RPMI media containing 10% FBS. Invasion inserts containing cell suspensions in media with 0.1% FBS were then carefully transferred into the wells containing media with 10% FBS. The plates were incubated for 22 h at 37°C/5% CO_2_ to allow the cells to invade from the upper chamber through Matrigel to the lower chamber of inserts. The non-invading cells on the upper chambers of inserts were quickly scrubbed off using cotton swabs and the invading cells on the lower chambers of inserts were fixed with 3.7% formaldehyde in PBS and then stained with 1% crystal violet. Invading cells were imaged using an Olympus IX70 microscope, quantified using the NIS-Element Software, and analyzed using GraphPad Prism 5.0.

### PCAIs inhibition of spheroid invasion

For these assays we adapted a protocol described by Vinci et al. [56]. H1299 lung cancer cells were seeded onto 96U Nunclon Sphera plates (Thermo Scientific, Waltham, MA) at a density of 2.5 × 10^4^ cells/mL in complete media. The cells were incubated at 37°C/5% CO_2_ for 48 h to allow for the formation of compact spheroids. After compact spheroids had been formed, a vial of BD Matrigel (Corning, Bedford, MA) was thawed on ice, and 450 μL volumes were added into each of eight pre-chilled micro-centrifuge tubes on ice. RPMI medium (50 μL) containing PCAIs was then added to each tube to yield final PCAIs concentrations of 0.0, 1.0, 2.0 or 4.0 μM of PCAIs in the Matrigel mixture. The Matrigel-PCAIs mixtures were gently mixed and then 100 μL aliquots of mixtures were transferred to 100 μL of spheroid suspension in the 96U plates. Final PCAIs concentrations in the Matrigel-PCAI-spheroid pool thus ranged from 0 - 2 μM. The 96U plate was incubated at 37°C/5% CO_2_ for 1 h to allow Matrigel to solidify while embedding the spheroids. After the Matrigel had solidified, it was overlaid with 100 μL of experimental media containing the corresponding concentrations of PCAIs (0 – 2 μM). At this juncture, bright-field images of the spheroids embedded in Matrigel were immediately obtained and this time point was designated as the 0 h time point. The 96U plate was then incubated (37°C/5% CO_2_) for an additional 72 h before the spheroids were re-imaged using a Nikon *Ti* Eclipse Microscope (4X magnification). The area of invasion was determined using the NIS-Element software. Data were analyzed using Prism GraphPad Prism 5.0.

### Cell survival and colony formation assay

The ability of NSCLC to survive and proliferate after exposure to PCAIs was tested. Cells were seeded in 6-well plates (Corning, Bedford, MA) at a density of 2.0 × 10^5^ cells/mL and incubated at 37°C/5% CO_2_ overnight. The next day, the cells were treated with varying concentrations of PCAIs (0 - 4 μM) and then incubated again at 37°C/5%. After 24 h post initial treatment, the cells were re-treated with equal amounts of PCAIs (0 - 4 μM) and incubated for an additional 24 h for a 48 h exposure. Following treatment, the cells were lifted, counted, and diluted so that cell suspensions containing 0.5 × 10^3^ or 1.0 × 10^3^ cells in 2 mL of RPMI medium were plated in triplicates into new 6-well plates. These were then incubated at 37°C/5% for 10 -12 days. Colonies were fixed with a 7:1 mixture of methanol to acetic acid and stained with 1% crystal violet. Digital images of the colonies were captured, counted, and analyzed using GraphPad Prism 5.0.

### PCAIs inhibition of spheroid formation

H1299 cell suspensions (2.5 × 10^4^ cells/mL) in treatment media containing varying concentrations of PCAIs (0 - 2 μM) were seeded into the wells of a 96U Nunclon Sphera plate (Thermo Scientific, Waltham, MA). After 24 h, equivalent amounts of PCAIs were supplemented to each well of the 96U plate. The ability of the cells to form viable spheroids was examined at 48 h post exposure to PCAIs. The viabilities of the spheroids was determined by staining them with AO/EB (5 μg/mL) solution and then capturing fluorescent and bright-field images. The ratios of the fluorescent intensities of AO over EB for the respective PCAIs concentrations used were computed and the data was graphed using GraphPad Prism 5.0.

### Phalloidin staining assay

The effect of PCAIs on F-actin assembly and organization was investigated using Phalloidin-iFluor 488 (Abcam, Cambridge, MA). H1299 cell suspensions containing 1.0 × 10^4^ cells/mL were seeded onto glass coverslips that were placed into the wells of a 24-well plate. The cells were then incubated overnight to adhere onto the coverslips. Once attached, complete media was removed and replaced with treatment media containing 0 μM, 5 μM or 10 μM of the PCAIs or the non-farnesylated analog, NSL-100, and incubated for 24 h. The cells were then fixed onto the coverslips by treating with 3.7% formaldehyde, permeabilized with 0.1% Triton X-100 and stained with Phalloidin-iFluor 488. The coverslips were mounted onto microscope slides using mounting media containing DAPI and fluorescent images of the F-actin cytoskeleton were captured with a Nikon *Ti* Eclipse microscope at 40X magnification. The number of lamellipodia and filopodia were quantified using NIS-Element Software and graphed using GraphPad Prism 5.0.

### Statistical analysis

All results are the means ± SEM. Symbols on graphs represent statistical comparisons to the corresponding controls following analysis of variance with Dunnett's post-hoc multiple comparisons tests or by Student's t-test as indicated in the figure legends. *p* values less than 0.05 were considered to be significant.

## SUPPLEMENTARY MATERIALS FIGURES


