# Supplementary material for: Polyisoprenylated cysteinyl amide inhibitors disrupt actin cytoskeleton organization, induce cell rounding and block migration of non-small cell lung cancer
Source: Oncotarget. 2017 Mar 7;8(19):31726–44. doi: 10.18632/oncotarget.15956 (PMC5458243; doi:10.18632/oncotarget.15956)
Supplement: Supplementary file 1 [file oncotarget-08-31726-s001.pdf]

Polyisoprenylated cysteinyl amide inhibitors disrupt actin cytoskeleton organization, induce cell rounding and block migration of non-small cell lung cancer

SUPPLEMENTARY MATERIALS

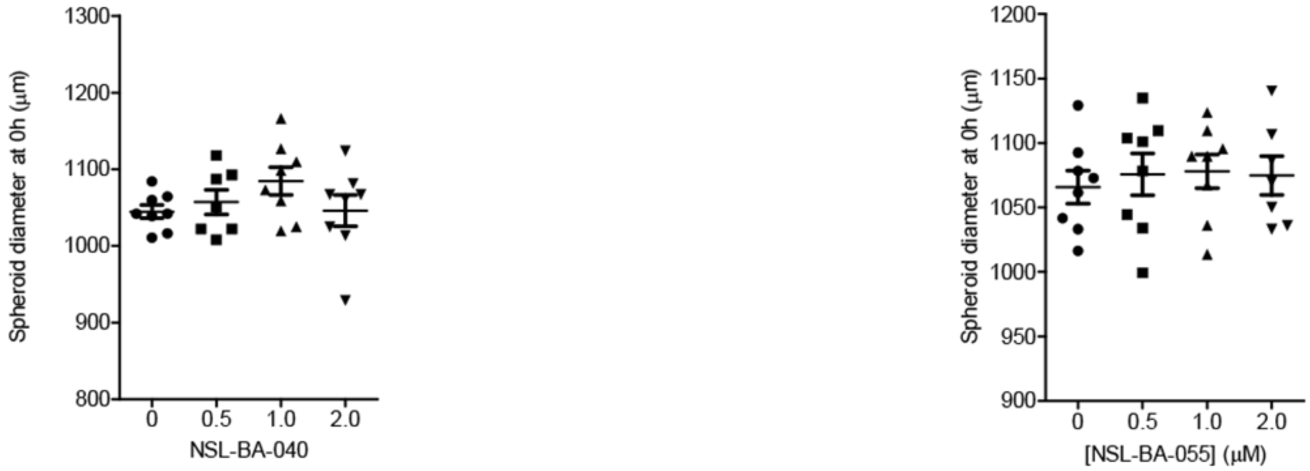

Supplementary Figure 1: H1299 Spheroids of similar diameter were used for spheroid invasion assay. Spheroids of comparable diameter were generated by plating 5×10^3 cells/well into each of the wells of a 96-well Nunclon Sphera plate. After 48 h incubation, compact spheroids had formed. Spheroid diameters were measured using the NIS-Element software before they were embedded into Matrigel as described in Figure 2C. The graphs indicate the diameter of spheroids that were exposed to acetone only, or to the PCAIs.
